# Breeding Tools for Assessing and Improving Resistance and Limiting Mycotoxin Production by *Fusarium graminearum* in Wheat

**DOI:** 10.3390/plants11151933

**Published:** 2022-07-26

**Authors:** Sandiswa Figlan, Learnmore Mwadzingeni

**Affiliations:** 1 Department of Agriculture and Animal Health, Science Campus, University of South Africa, Corner Christiaan De Wet and Pioneer Avenue, Private Bag X6, Florida 1709, South Africa; 2 Seed Co Limited, Rattray Arnold Research Station, Chisipite, Harare P.O. Box CH142, Zimbabwe; mwadzingenil@yahoo.com

**Keywords:** contamination, health, infection, molecular techniques, selection

## Abstract

The recently adopted conservation and minimum tillage practices in wheat-production systems coupled with the concomitant warming of the Earth are believed to have caused the upsurges in Fusarium head blight (FHB) prevalence in major wheat-producing regions of the world. Measures to counter this effect include breeding for resistance to both initial infection of wheat and spread of the disease. Cases of mycotoxicosis caused by ingestion of wheat by-products contaminated with FHB mycotoxins have necessitated the need for resistant wheat cultivars that can limit mycotoxin production by the dominant causal pathogen, *Fusarium graminearum*. This manuscript reviews breeding tools for assessing and improving resistance as well as limiting mycotoxin contamination in wheat to reflect on the current state of affairs. Combining these aspects in wheat research and development promotes sustainable quality grain production and safeguards human and livestock health from mycotoxicosis.

## 1. Introduction

Breeding wheat for Fusarium head blight (FHB) resistance involves systematic genetic manipulation of the crop to incorporate superior biochemical and morpho-physiological traits that safeguard it against the damaging effects of the dominant causal species, *Fusarium graminearum.* Infection of crops by *F. graminearum* does not only reduce yield, but also exposes the grain to contamination by mycotoxins. Mycotoxin contamination in grain crops intended for processing food, feed and beverages often results in the accumulation of these toxic fungal metabolites in foodstuffs, causing health hazards to both human beings and livestock. *F. graminearum* species complex infects grain crops including wheat, barley and maize. Breeding for resistance against FHB aims to reduce the impact of the pathogen on crop yield as well as mycotoxin contamination in infected grain. Various strategies for breeding against Fusarium head blight have been embarked on because resistance against the disease is multigenic and is further confounded by the large influence of genotype by environment interactions [1,2]. Resistance against FHB is conferred by more than 250 quantitative trait loci (QTL) distributed across the entire chromosome cascade of the wheat genome [3,4,5]. To effectively compart the negative effects of the disease, strong background knowledge is needed on various aspects including the importance of FHB as a grain disease, mycotoxin contamination of infected grain, breeding strategies to reduce mycotoxin contamination in grain as well as the tools used to assess and limit mycotoxin contamination during breeding, selection and the entire wheat value chain.

Fusarium head blight, also known as ‘scab’, is a wheat disease that is mainly caused by the fungal complex called *F. graminearum* Schwabe (teleomorph *Gibberella zeae* Schwein. Petch). It is one of the most common diseases affecting bread wheat (*Triticum aestivum* L.) around the world. Epidemics of FHB occur in cycles of four or five years worldwide [6] and in shorter periods under favorable conditions, particularly where no-till or minimum tillage practices, high humidity and/or high temperature coincide with early flowering to the soft dough stages of susceptible wheat cultivars. In addition to the enormous grain yield losses, *F. graminearum* infection is associated with the accumulation of mycotoxins that put the health of human beings and livestock consuming infected grain at risk [7]. Ingestion of huge amounts of mycotoxin contaminated grain may lead to mycotoxicosis, which under severe circumstances may cause death. It is important to note that there is very high genetic variability of *F. graminearum* species, which results in high resilience and complicates efforts towards breeding for FHB resistance due to genotype by isolate and isolate by environment interactions [8].

*F. graminearum* produces two groups of toxins, namely zearalenone and trichothecenes. Zearalenone (previously referred to as F-2 toxin) is one of the most prevalent estrogenic mycotoxins produced through the polyketide pathway. This mycotoxin is denoted as 6-[10-hydroxy-6-oxo-*trans*-1-undecenyl]-B-resorcyclic acid lactone. Zearalenone derives its name from *Gibberella zea* and, resorcyclic acid lactone because of the C-1′ to C-2 ‘double bonds. The ‘-one’ denotes its ketone group [9]. The toxicity of zearalenone is through binding to estrogen receptors ending up in estrogenicity, occasionally causing hyperestrogenism in livestock and human beings, especially women. Eventually, the toxicity of zearalenone may lead to myelofibrosis, reproductive system disorders, cancers, skeletal malformations and weakening [10], nervous disorders [11] and various other physiological malfunctions. Trichothecenes, on the other hand, are chemically tricyclic sesquiterpenes, which have double bonds at the C-9, 10 position and a C-12, 13 epoxy functional group. The most common contaminants of cereals are type-A and type-B trichothecenes [12,13]. Type-A trichothecenes are different from type-B by the absence of a carbonyl group at C-8 and hydroxylation at C-7. Type-A mycotoxins include diacetoxyscirpenol, T-2 and HT-2 toxins while type-B trichothecenes include fusarenone-X, nivalenol and deoxynivalenol. The effects of trichothecene ingestion through contaminated foodstuffs by animals and human beings include diarrhea, vomiting and death when the toxicosis is severe.

Various national and multi-national organisations have drafted guidelines on food safety to ensure that consumers are safe from the risks of eating contaminated food. The regulating bodies include the European Food Safety Authority, Codex Alimentarius and the USA Food and Drug Administration. Realizing that it is not possible to produce mycotoxin-free wheat grain, the regulating bodies have set threshold limits, which are practically attainable to reduce the incidence of mycotoxins in wheat products and other foodstuffs. The threshold regulations mainly protect the health of animals and human beings from the dangers caused by mycotoxins. Contamination of wheat with mycotoxins occurs during infection by mycotoxin, producing fungi such as *F. graminearum*, and further toxin accumulation may occur postharvest during grain storage [14,15,16,17]. Various interventions are necessary to limit mycotoxin contamination of the wheat grain by *F. graminearum* mycotoxins.

FHB can be managed using various strategies including cultural, biological and chemical control methods as well as breeding for resistance against the disease. Wheat production has thrived for ages through selection for superior traits and painstaking efforts to incorporate disease resistance. With increased efforts to incorporate FHB resistance into wheat, disease incidence and the spread of infection decrease, resulting in a subsequent reduction in mycotoxin contamination. Moreover, resistance may be specific to reduce mycotoxin production by the infecting *F. graminearum*. Various breeding strategies are being embarked upon to ensure minimal mycotoxin contamination of wheat grain. It is also important to develop laboratory tools to assist the selection of wheat varieties that suppress mycotoxin production as well as to ensure compliance with wheat grain safety standards. This review discusses these aspects beginning with various breeding strategies employed against FHB. Emphasis has been put on traditional breeding strategies, new techniques of resistance breeding and tools for monitoring mycotoxin levels in the harvested wheat grain.

## 2. Resistance against Fusarium Head Blight in Wheat

Resistance to FHB is categorised into various types of which the most prominent ones are type I and type II [18]. Type I refers to the resistance against initial infection and is exhibited by the ability of the cultivar to create a barrier to initial entry of the pathogen into the plant. On the other hand, Type II resistance is resistance to the spread of the pathogen after it has gained entry into the plant. The later type of resistance is more stable. Type I and II resistance can be tested under both field and artificial environments [19]. Usually, screening for resistance against FHB takes place in the advanced generations like F_4_ onwards [20]. Select breeding lines are chosen and are artificially inoculated with the pathogen isolate(s)/races(s) to screen for resistance [21]. Assessment of resistance to FHB is done through generally visualizing discolouration of the spikes and by precisely assessing the intensity and number of affected grains. Affected grain may have a pinkish discolouration, sometimes with a chalky appearance. Assessment covers both the proportion of kernels that are diseased and the level of mycotoxins in the affected grain [22]. Resistance against mycotoxin accumulation is called type III resistance, which requires special tools for assessment, unlike type I and II which can be assessed visually. Both type I and type II resistance have indirect effects on toxin accumulation, but resistance to toxin accumulation, type III resistance, still has to be a targeted breeding objective on its own. Generally, genotypes to be used as donors of resistance in FHB breeding programmes and ultimate varieties must (1) resist initial infection (type I), (2) limit the pathogen spread in infected spikes (type II), (3) reduce mycotoxin accumulation in the grain (type III)), (4) resist kernel damage (type IV) and (5) tolerate the presence of the disease without much yield penalty (type V) [19]. Knowledge of the genetic basis underlying these observable types of resistance is slowly being demystified through advanced biotechnology and genetics.

## 3. Breeding Focus against Fusarium Head Blight

With the development of settlements for human beings and crop domestication, early farmers selected plants that had desirable traits and the resulting gene pool formed the basis of today’s domesticated crops. Natural selection for superior agronomic traits was accelerated by the active mating and selection of offspring with desirable traits. Crops progressively improved, hence, huge monoculture practices were established to what has become modern agriculture. Wheat is one of the crops that has been extensively bred over the years leading, notably, to the Green Revolution of the 1960s. After a prolonged period of painstaking breeding efforts, Dr. Norman Borlaug, the Father of the Green Revolution, developed high yielding wheat varieties in India and Pakistan, a move that averted massive hunger. Despite this milestone, various diseases continue to threaten the crop, particularly wheat rusts and Fusarium head blight. Breeding for disease resistance continued to protect yields of high yielding varieties, among other control strategies. The wheat disease resistance breeding strategy at the International Centre for Maize and Wheat Improvement (CIMMYT) systematically grouped breeding needs of various regions in the world into mega-environments [23]. Breeding for resistance against FHB falls within the needs of mega-environment 2, which is characterized by high rainfall. China has been a significant source of resistance to FHB and hundreds of wheat lines carrying resistance have been shared with CIMMYT. Among the Chinese lines that carry FHB resistance are Sumai#3, Shanghai#5, Suzhoe#6, Yangmai#6, Wuhan#3 Ning 7840, and Chuanmai 18, which have been developed using traditional breeding methods. Genes for resistance against FHB are mostly additive, requiring a meticulous programme for resistance incorporation and selection [24].

Genetic variation for FHB resistance breeding is large. Therefore, there is a wide pool of sources of resistance. This makes it easy for resistance to be incorporated into wheat with options from exotic and native sources. However, Asian sources of resistance against FHB such as the Chinese spring wheat, Sumai#3, are prominently used worldwide. Resistance to FHB is mostly additive, being controlled by the effects of multiple genes. Quantitative trait loci controlling FHB across all 21 bread wheat chromosomes have been mapped and identified, with just a few validated and used in breeding [4,5,25]. These QTL are prevalent in Chinese genotypes derived from Sumai#3 and they contain *Fhb1*, *Fhb2*, as well as *Qfhs.ifa-5A* [26,27,28,29,30,31,32]. Nevertheless, other resistance QTL do exist outside of Sumai#3. The presence of *Fhb1* (Sumai#3) and *Qfhs.nau-2DL* (breeding line CJ9306), which confer resistance to both type II and type III resistance, are of particular interest. *Fhb1* improves the detoxification of deoxynivalenol (DON) to DON-3-glucoside [33]. *Qfhs.ifa-5A* confers type III resistance by suppressing mycotoxin accumulation. Although resistance to FHB acquired from sources such as Sumai#3 has been useful, its use has been moderate and therefore new sources of resistance are desperately needed, especially resistance to curb toxin accumulation in wheat infected with *F. graminearum*. The current shortfalls in breeding for resistance against FHB therefore require radical use of new technologies. These technologies will help to improve wheat productivity to meet the needs of the growing global population.

Wheat breeding programs against FHB also aim to reduce mycotoxin production by the infecting fungus *F. graminearum*. From a food safety concern, this is an important breeding objective to ensure that harvested grain is strictly below the mycotoxin threshold level. To breed for resistance against FHB, a reliable inoculation method is needed. This allows repeatable assessment of resistance to ensure selection of resistant lines under high and uniform disease pressure. It is also important to use a cocktail of isolates/races for inoculation to ensure selection for broad-spectrum or multi-race resistance, preferably using races prevalent in the area where the resistant cultivars will be released. Isolates that produce higher levels of DON, a type-B trichothecene, are found to be more aggressive and could be useful for effective selection for type III resistance [34,35,36,37,38,39]. Resistance of wheat to DON accumulation is acquired through the ability of the plant to degrade the mycotoxin, for example, the possession of a putative deoxynivalenol-glycosyl transferase that detoxifies DON [33,40]. Newer strategies for resistance breeding have been adopted over the years and progress has been made ever since the adoption of these technologies. Breeding programs that aim to limit DON production by *F. graminearum* in wheat have greatly benefited from these new technologies.

## 4. Traditional Crop Breeding against Fusarium Head Blight

Conventional breeding is a systematic hybridization and selection strategy aimed to release superior genotypes. In certain instances, the trait of interest is transferred from a wild relative of the crop to be improved and this is termed wide crossing. Breeding for disease resistance often takes a different strategy from conventional breeding for complex agronomic traits such as yield. There has to be a source of resistance, which donates the resistance gene/genes to the recipient genotype containing most of the desirable agronomic traits, except for the resistant gene(s) of interest. In such a scenario, backcross breeding, which is the most prominent classical breeding technique against plant diseases, is used to recover most of the recipient genotype’s genome. In certain instances, the resistance incorporated into a cultivar against FHB may be race-specific, though in most cases it is race non-specific. It is always important to adopt a clear resistance breeding strategy so that broad-spectrum and durable resistance may be incorporated into the cultivar. When using traditional breeding techniques, it is critical to select effectively in the early generations for FHB resistance; otherwise the promising gene combinations are lost irretrievably [41]. Thus, the selection efficiency increases when the breeding method can be used to select successfully in the early generations of selection [41]. Following the vast research investments that were put towards FHB resistance, backcross breeding is no longer sorely classical but is now fused with various molecular marker techniques for effective and timely selection as well as gene and QTL introgression.

## 5. Molecular Breeding Techniques

The use of resistant cultivars remains a valuable tool for the control of FHB. It therefore remains imperative to intensify breeding efforts and optimize breeding and selection strategies for resistance against FHB and mycotoxin production. The development and improvement, in recent years, of molecular techniques like real-time polymerase chain reaction (PCR), marker-assisted selection, marker-assisted QTL backcrossing, next generation sequencing technologies and genetic engineering, are boosting research on FHB resistance and its associated mycotoxicosis. Screening for resistance against FHB usually takes place in advanced generations like F_4_ onwards when select breeding lines are chosen and artificially inoculated with the pathogen to screen for resistance [42]. This task is very laborious and requires time for completion. In this case, advanced molecular techniques are required to monitor levels of inoculation, to select for resistance in genotypes to be used as parents in breeding for resistance to FHB and to introgress resistance genes into elite genotypes. These molecular tools are therefore useful in wheat pre-breeding and breeding against FHB.

### 5.1. RNA Interference to Reduce Mycotoxin Contamination in Fusarium graminearum Infected Wheat

The discovery of more sophisticated biotechnological approaches such as ribonucleic acid (RNA) interference (RNAi) offers new transformation opportunities to enhance resistance against *F. graminearum* and other invading wheat pathogens [43]. This is achieved through induced silencing of target virulent genes. RNA interference is an essential cellular system involved in gene regulation and protection of eukaryotes against infection by viruses [44]. It is an important systematic mechanism that can be employed to fight mycotoxigenic plant pathogenic fungi like *F. graminearum*. RNAi post-transcriptionally converts double stranded RNA molecules into short-stranded RNA duplexes of about 21 to 28 nucleotides often termed short interfering RNAs (siRNAs), which then cleaves to complimentary mRNA, effecting gene silencing or regulation [45,46,47,48]. RNA interference pathways are often triggered by the presence of viral RNAs providing gene regulated defense against specific RNA viruses. In this case, the mechanism will be termed virus-induced gene silencing (VIGS), whose success is highly dependent on designing effective vectors that will produce complementary siRNA species, efficient uptake of siRNAs by the fungus and amplification of the silencing effect within the target organism [43]. Silencing of target genes has recently been proved to be effective against plant pathogenic fungi [49] and has been demonstrated on *Puccinia* in wheat among other crop species and their respective fungal pathogens. Machado et al. [50] reviewed the recent advances in RNAi-mediated FHB control and suppression of mycotoxin contamination in a number of cereals. This involves the use of the barley stripe mosaic virus (BSMV) vector. *P. striiformis* genes were also observed to be silenced using the host-induced RNA interference mechanism [51]. In a more recent study, Cheng et al. [52] reported that wheat resistance against pathogenic fungi can be improved through RNAi sequences originating from chitin synthase (Chs) 3b gene originating from *F. graminearum.* These sequences are used for host-induced silencing of the chitin synthase gene in plant pathogenic fungi. This is one of the techniques that holds future promise for the incorporation of resistance against *F. graminearum* in wheat.

### 5.2. Gene Transfer in General and Specifically against Fusarium Head Blight

Gene transfer technologies that insert foreign genes in plants are another molecular breeding strategy with potential to enhance wheat resistance to FHB [53]. These technologies include particle bombardment or biolistic transformation and *Agrobacterium*-mediated genetic transformation [54]. The former bombards deoxyribonucleic acid (DNA)-coated gold or tungsten micro-projectiles into the target crop’s genome using a particle gun, thereby inserting foreign genes. The later technique uses *A. tumefaciens* as a vector that copies and transfers the transfer DNA (T-DNA) molecules on a tumour-inducing (Ti) plasmid into the nucleus of target plant cells, thereby incorporating foreign DNA that is eventually inserted and becomes part of the plant genome. *Agrobacterium* transformation, however, works effectively with selected plant species, and inserts mostly three genes, including two T-DNA molecules and a selectable marker per transformation construct [55]. Biolistic transformation non-randomly targets AT-rich regions with matrix attachment region (MAR) motifs that are nuclear matrix prone eukaryotic DNA elements [56,57]. The MARs create open chromatin, allowing the host plant genome to be accessible to transgenes. An advantage shared by both *Agrobacterium* transformation and biolistic transformation is that they can integrate two trans-genes into the target host genome [58].

The *Agrobacterium*-mediated transformation stages involve initiation, which includes identification, isolation and insertion of the gene of interest into a suitable functional construct consisting of the gene expression promoter, gene of interest, selectable marker and codon modification. This is followed by *Agrobacterium*-mediated transformation or bacterium-to-plant transfer and finally nucleus targeting [59,60,61]. During gene transfer within the plant cell, the transformed *Agrobacterium* facilitates the transfer of T-DNA molecules into the plant genome, then the transgene is randomly incorporated into the plant chromosome. Integration of T-DNA into the plant DNA sequence is then facilitated by non-homologous end-joinings.

Transfer of foreign genes that enhance FHB resistance into wheat is a viable alternative which has, in recent years, been used extensively to increase not only the crops’ genomic variability, but also the fitness of wheat against *F. graminearum*. Among first genes to be transferred since 1992 was the *Bar* gene used as a selective marker and various others including the *TaPIMP1* gene [62], the *Yr10* gene [63] and the *TcLr19PR1* gene [64]. Various genes that encode pathogenicity related proteins (PR proteins) could be the new sources of wheat resistance against FHB. These PR proteins are defensins, which have a broad range of antifungal properties [65]. Defensin RsAFP_2_ with growth inhibitory characteristics against *F. graminearum* was incorporated into variety Yangmai 12 using biolistic particle bombardment [66]. The success of the transformation was confirmed using PCR and Southern blot analysis. Expression of the *RsAFP_2_* genes in transformed wheat lines was confirmed using RT-PCR and Western blotting. Disease resistance was assessed, and the transformed lines showed resistance against *F. graminearum* compared to the untransformed control lines [66]. The low transformation efficiency using the biolistic particle bombardment, however, warrants the need for other gene transformation techniques alongside. *Agrobacterium*-mediated transformation is one such technique that has been used successfully to introduce foreign genes into the wheat plant with improved transformation efficiency.

In one effort, chitinase and #beta#-1,3-glucosanase genes were transformed into wheat to improve resistance against FHB. The transformation of chitinase and #beta#-1,3-glucosanase genes (constructed into binary vector pCAMBIA3301) was mediated by *Agrobacterium* and the resultant transgenic lines showed resistance against FHB in the field [67]. Transformation of plant cells with exotic genes mediated with *Agrobacterium* is the initial step in introducing genes into plant cells that generate into adult plants capable of producing normal seeds. However, this process is difficult with wheat because of its complex hexaploid genome. Therefore, a more efficient protocol for wheat transformation called, ‘Pure Wheat’, was introduced [68]. This technique has renewed hope in accelerating transgenic wheat plants with superior traits such as FHB resistance and its associated ability to limit mycotoxin production.

### 5.3. Genome Editing for FHB Resistance

Major improvements in wheat will likely be brought about by genome editing, which promises to supersede the traditional random mutagenesis and conventional breeding. Genome editing technologies include the clustered regularly interspaced short palindromic repeat-associated endonucleases (CRISPR/Cas) technique, which is gaining much popularity, and other sequence-specific nucleases (SSNs) such as the transcription activator-like effector nucleases (TALENs) and zinc-finger nucleases (ZFNs). These technologies offer the benefits of gene knock-out, knock-in, replacement, activation and DNA repair [69,70,71,72]. Among these genome editing technologies, the CRISPR/Cas technology seems to hold more promise with regards to FHB resistance. The Cas nuclease system has been used with success in understanding fungal biology, with various reports in *Neurospora crassa* [73], *Aspergillus* spp. [74,75], *Penicillium chrysogenum* [76], *Alternaria alternata* [77], *Pyricularia oryzae* [78] and *Ustilago maydis* [79]. Following on these milestones, a Cas9-based genome editing system was established in *F. graminearum* [80] and hopefully this study will generate leads to a breakthrough in *F. graminearum* control.

Several research groups have made concerted efforts to develop transgenic and mutagenic lines that confer resistance to FHB. Table 1 summarizes some of the genes that have been manipulated in wheat, barley, *Brachypodium* and *Arabidopsis* that were manipulated through advanced technologies and proved to confer reduced *F. graminearum* infection and DON accumulation. However, much effort is still needed to link the various research institutions with public and private seed companies to ensure that research and development are aimed at variety release to benefit farming communities in FHB prone areas. This effort should involve pre-commercial field-testing activities including multi-environmental trials and end-use quality analysis.

### 5.4. Association Mapping to Find FHB Molecular Markers

Molecular breeding and selection for FHB resistance in wheat have largely benefited from association mapping of putative QTL through associating phenotypic reactions to genotypes. Currently, high-density wheat 90 K single nucleotide polymorphism (SNP) assays are being used in genome-wide association (GWAS) studies aimed to dissect the genetic basis of resistance to Fusarium head blight in wheat breeding populations [92]. Association mapping studies have enabled the discovery of several loci associated to the resistance to FHB spread and DON accumulation. Alternative to the GWAS approach, candidate-gene association mapping can be used by targeting associations of pre-specified FHB resistance genes and the observed phenotypic reaction [93]. A recent GWAS study identified 16 significant SNPs associated with Fusarium-damaged kernels and DON levels on wheat chromosomes and suggested that FHB severity can even be reduced by small-effect QTL [94]. Such studies form the basis of maker-assisted selection and marker-based gene and/or QTL introgression by identifying putative markers linked to genetic regions controlling particular traits. Quality phenotypic data, often with high heritability from multi-environmental trials, is required for effective association studies.

All these advanced technologies that can be employed to enhance FHB resistance have their own advantages and disadvantages when compared to traditional breeding methods. Table 2 highlights some of these pros and cons to guide future research. Generally, this indicates that the recent technologies can not completely be divorced from all aspects of traditional breeding, particularly phenotyping or field testing to account for the expression of introduced genes under real production conditions and assessing the ultimate impact on final yield.

## 6. Tools to Assist Breeding for Resistance against FHB and Mycotoxin Contamination

Laboratory analytical tools are useful to assess toxin accumulation in wheat infected with *F. graminearum.* These tools can be used in breeding programmes to assess if resistance to mycotoxin accumulation by *F. graminearum* is incorporated and in monitoring the safety of food products made from wheat grain. To incorporate Fusarium head blight resistance in wheat, various assessment methods are employed for each breeding objective. Resistance against pathogen penetration and resistance against disease spread after initial infection can be monitored visually. Monitoring resistance against mycotoxin accumulation requires specialized equipment that is able to detect even trace amounts of the mycotoxins. For the purposes of the current review, real-time PCR, chromatography and mass spectrometry-based approaches are discussed as tools to assist selection.

### 6.1. Real-Time PCR

Inoculation with *F. graminearum* and then determining the quantity of the inoculum is done by real-time PCR. Real-time PCR is important for diagnoses using species-specific primers to detect a suspect pathogen and for quantifying pathogen titre in infected kernels [95,96,97,98]. The technique has the potential to unpack the gene expression in response to FHB infection through monitoring transcriptome expression patterns within specific plant tissue after inoculation. Newer genomic technologies, such as genome-wide single polymorphism mapping, genome sequencing, microarrays and RNA sequencing, have been instrumental in identifying genotypes with FHB resistance. These techniques have also been useful in identifying QTL, linking resistance with other phenotypic traits as well as detecting and validating diagnostic markers.

### 6.2. Chromatography and Mass Spectrometry-Based Approaches to Assist Selection

Regulatory standards with threshold prescriptions for wheat products such as the Codex Alimentarius Commission 2015 require that there are monitoring procedures to quantify the DON toxin in harvested wheat grain and grain products. Chromatography and mass spectrometry-based techniques become handy in such circumstances to ensure safety of wheat products in the market. Notably, high performance liquid chromatography (HPLC) is commonly used for separation, identification, and quantification of mycotoxin levels in flour, food and feed mixtures. Other techniques include gas chromatography–mass spectrometry (GC-MS) and thin-layer chromatography (TLC), which are also effective for early detection and quantification of DON in wheat. Equally important is the use of these quantitative techniques in screening breeding material and donor lines to be used in breeding against FHB, especially for type II resistance. Chromatography and mass spectrometry have been useful in identifying mycotoxin contaminants of wheat [96] and mycotoxin accumulation [99]. Because of their ability to detect and quantify contaminants and trace elements, chromatography and mass spectrometry-based techniques are useful in routine monitoring of grain safety to ensure compliance to prescribed standards. This could be the extension of the use of these techniques beyond research. With these state-of-the-art tools, breeding and selection of FHB resistant genotypes are becoming more efficient and reliable data are being produced on resistance to infection and mycotoxin contamination in the wheat grain.

## 7. Conclusions

The safety of wheat products is essential to ensure that human and animal lives are not endangered. Mycotoxins produced by the wheat-infecting *Fusarium graminearum* pathogen pose serious health risks to animals and human beings. It is therefore of the utmost importance to breed wheat varieties that are able to limit the accumulation of mycotoxins in wheat kernel that have been infected with *F. graminearum*. Traditional breeding techniques have been utilized to incorporate resistance against *F. graminearum* from resistance sources such as Sumai#3. However, the limitations of traditional plant breeding require integration of new and more sophisticated methods for cultivar improvement to fast-track *F. graminearum* resistance breeding. These techniques will also bolster resistance against mycotoxin accumulation. Clustered regularly interspaced short palindromic repeat-associated endonucleases (CRISPR/Cas) as well as RNA interference are some of the advanced tools that have revolutionized crop improvement efforts. Various molecular techniques like real-time PCR and biochemical analytical tools such as chromatography and mass spectrometry are also useful for detecting levels of infection by *F. graminearum*, and their use remains relevant for the future.

## Figures and Tables

**Table 1:** Genetic transformation to enhance resistance against *Fusarium graminearum* causing Fusarium head blight in wheat and other cereals.

Crop	Technology	Gene Involved	Effect on Transformed Line	Reference
Wheat	Gene silencing	Chitin synthase *ChS3B*	Enhanced combined type I and II resistance against FHB by targeting chitin biosynthesis	[52]
Barley	Gene silencing	*FgCYP51A* and *FgCYP51B*	Reduced fungal growth by targeting Sterol biosynthesis	[81]
*Brachypodium distachyon*	Gene silencing	*Fg00677*, *Fg08731* and *CYP51*	Improved FHB resistance by silencing the genes through inhibiting *CYP51A*, *CYP51B*, and *CYP51C* genes and essential protein kinase biosynthesis	[82]
Wheat	Deletion mutation	*TaHRC*	Enhanced FHB resistance by silencing the gene that encodes a nuclear protein conferring FHB susceptibility	[83]
Wheat	Overexpression	*Barley HvUGT13248*	Decreased DON content in flour by increasing detoxification	[84]
*Arabidopsis*	Gene silencing	*CYP51*	Restricted fungal infection and reduced virulence by targeting Ergosterol biosynthesis	[85]
Barley	Gene silencing	*CYP51*	Restricted fungal infection and reduced virulence by targeting Ergosterol biosynthesis	[85]
Wheat	Overexpression	*AtLTP4.4*	Supressed DON induced reactive oxygen species and plant stress from infection	[86]
Wheat	Gene silencing	*FgSGE1*, *FgSTE12* and *FgPP1*	Reduced infection and DON accumulation by targeting DON biosynthesis, fungal penetration structure formation and essential phosphatase	[87]
Wheat	Epigenetic regulation of gene expression	Several	Reduced FHB severity and DON accumulation through methylation	[88]
Wheat	Trans-gene expression	*Bradi5g03300 UGT*	Conferred resistance both to initial infection and to spike colonization and reduce mycotoxin content	[89]
Wheat	Mutation/Deleation involving the 3′ exon	histidine-rich calcium-binding-protein gene	Resistance to FHB spread	[90]
Wheat	Trans-gene expresion	*HvUGT13248*	Increased resistance against *Fusarium graminearum*	[91]

**Table 2:** Pros and cons of using traditional breeding methods against using recent technologies.

Aspect	Traditional Methods	Recent Technologies
	**Pros**	**Cons**
Field expression of genes	Reliably confirmed each season	Gene may be present but not expressed as desired in the field [41]
Variety release	Often targeted towards FHB resistant variety release and commercialization across multiple environments	Mostly limited to research and laboratory experiments under controlled environments
Skills and reaserch facilities	Readily available	Still limited with most institutions outsourcing and licencing the technologies
Selection methods	Well established breeding and selection procedures	Procedures mostly still being developed and improved
Acceptability	Widely accepted	Some technologies like gene transformation are not widely accepted by policy makers and consumers
	**Cons**	**Pros**
Time utilization	Takes long–up to 12 years to release a variety	Significantly reduced time depending on technology
Cost	Costly in terms of time and resources allocated to release a variety	Relatively cheap since the costs are concentrated over short space of time and less resources required
Environmental influence	FHB expression can be influenced by the environment during phenotyping [23]	Tracking of genes and transgenes at molecular level is more reliable
Space required	Several hectors of land are often required to handle breeding nurseries	Conversion and transformation often need lab and greenhouse space
Foreign genes	Restricted to the use of plants of the same genius or species (cross compatible)	FHB resistant genes can be transferred from different plant or micro species without fertilization barriers [53]
QTL conferring FHB resistance	Difficult to detect and transfer	Easy to detect and transfer [92,93,94]
Pyramiding and stacking multiple genes	Difficult	Easier with genetic engineering [87]

## Data Availability

Not applicable.

## References

[1] Kumar S., Saharan M.S., Panwar V., Chatrath R., Singh G.P. (2019). Genetics of Fusarium head blight resistance in three wheat genotypes. Indian J. Genet..

[2] Li H., Zhang F., Zhao J., Bai G., Amand P.S., Bernardo A., Ni Z., Sun Q., Su Z. (2022). Identification of a novel major QTL from Chinese wheat cultivar Ji5265 for Fusarium head blight resistance in greenhouse. Theor. Appl. Genet..

[3] Zhang J., Gill H.S., Brar N.K., Halder J., Ali S., Liu X., Bernardo A., St Amand P., Bai G., Gill U.S. (2022). Genomic prediction of Fusarium head blight resistance in early stages using advanced breeding lines in hard winter wheat. Crop J..

[4] Liu S., Hall M.D., Griffey C.A., McKendry A.L. (2009). Meta-analysis of QTL associated with Fusarium head blight resistance in wheat. Crop Sci..

[5] Buerstmayr M., Steiner B., Buerstmayr H. (2020). Breeding for Fusarium head blight resistance in wheat—Progress and challenges. Plant Breed..

[6] Figueroa M., Hammond-Kosack K.E., Solomon P.S. (2018). A review of wheat diseases—A field perspective. Mol. Plant Pathol..

[7] Mielniczuk E., Barbara S.-B. (2020). Fusarium head blight, mycotoxins and strategies for their reduction. Agronomy.

[8] de Arruda M.H.M., Zchosnki F.L., Silva Y.K., de Lima D.L., Tessmann D.J., Da-Silva P.R. (2021). Genetic diversity of *Fusarium meridionale*, *F. austroamericanum*, and *F. graminearum* isolates associated with Fusarium head blight of wheat in Brazil. Trop. Plant Pathol..

[9] Sheini A. (2020). Colorimetric aggregation assay based on array of gold and silver nanoparticles for simultaneous analysis of aflatoxins, ochratoxin and zearalenone by using chemometric analysis and paper based analytical devices. Microchim. Acta.

[10] Zinedine A., Soriano J.M., Molto J.C., Manes J. (2007). Review on the toxicity, occurrence, metabolism, detoxification, regulations and intake of zearalenone: An oestrogenic mycotoxin. Food Chem. Toxicol..

[11] Venkataramana M., Chandra Nayaka S., Anand T., Rajesh R., Aiyaz M., Divakara S.T., Murali H.S., Prakash H.S., Lakshmana Rao P.V. (2014). Zearalenone induced toxicity in SHSY-5Y cells: The role of oxidative stress evidenced by N-acetyl cysteine. Food Chem. Tox..

[12] Schollenberger M., Müller H.M., Rüfle M., Suchy S., Plank S., Drochner W. (2006). Natural occurrence of 16 Fusarium toxins in grains and feedstuffs of plant origin from Germany. Mycopathologia.

[13] Vanhoutte I., Audenaert K., De Gelder L. (2016). Biodegradation of mycotoxins: Tales from known and unexplored worlds. Front. Microbiol..

[14] Chammem N., Issaoui M., De Almeida AI D., Delgado A.M. (2018). Food crises and food safety incidents in European Union, United States, and Maghreb Area: Current risk communication strategies and new approaches. J. AOAC Int..

[15] FAO, WHO (1995). Codex General Standard for Contaminants and Toxins in Food and Feed.

[16] EC (2006). Setting Maximum Levels for Certain Contaminants in Foodstuffs and Amendments.

[17] FAO, WHO (2018). Codex Committee on Contaminants in Foods.

[18] Mesterhazy A. (2020). Updating the breeding philosophy of wheat to Fusarium head blight (FHB): Resistance components, QTL identification, and phenotyping—A review. Plants.

[19] Kubo K., Kawada N., Fujita M. (2013). Evaluation of Fusarium head blight resistance in wheat and the development of a new variety by integrating type I and II resistance. Jpn. Agric. Res. Q..

[20] Pumphrey M.O., Bernardo R., Anderson J.A. (2007). Validating the *Fhb1* QTL for Fusarium head blight resistance in near-isogenic wheat lines developed from breeding populations. Crop Sci..

[21] Francesconi S., Angelo M., Giorgio M.B. (2019). Different inoculation methods affect components of Fusarium head blight resistance in wheat. Phytopathol. Mediterr..

[22] Gaire R., Sneller C., Brown-Guedira G., Van Sanford D., Mohammadi M., Kolb F.L., Olson E., Sorrells M., Rutkoski J. (2022). Genetic Trends in Fusarium Head Blight Resistance from 20 Years of Winter Wheat Breeding and Cooperative Testing in the Northern USA. Plant Dis..

[23] Crespo-Herrera L.A., José C., Mateo V., Hans-Joachim B. (2022). Defining Target Wheat Breeding Environments. Wheat Improvement.

[24] Singh R.P., Rajaram S. (2002). Breeding for disease resistance in wheat. Bread Wheat: Improvement and Production.

[25] Löffler M., Schön C.C., Miedaner T. (2009). Revealing the genetic architecture of FHB resistance in hexaploid wheat (*Triticum aestivum* L.) by QTL meta-analysis. Mol. Breed..

[26] Zhao M., Leng Y., Chao S., Xu S.S., Zhong S. (2018). Molecular mapping of QTL for Fusarium head blight resistance introgressed into durum wheat. Theor. Appl. Genet..

[27] Bai G., Kolb F.L., Shaner G., Domier L.L. (1999). Amplified fragment length polymorphism markers linked to a major quantitative trait locus controlling scab resistance in wheat. Phytopathology.

[28] Anderson J.A., Stack R.W., Liu S., Waldron B.L., Fjeld A.D., Coyne C., Moreno-Sevilla B., Fetch J.M., Song Q.J., Cregan P.B. (2001). DNA markers for Fusarium head blight resistance QTLs in two wheat populations. Theor. Appl. Genet..

[29] Buerstmayr H., Lemmens M., Hartl L., Doldi L., Steiner B., Stierschneider M., Ruckenbauer P. (2002). Molecular mapping of QTLs for Fusarium head blight resistance in spring wheat. I. Resistance to fungal spread (Type II resistance). Theor. Appl. Genet..

[30] Buerstmayr H., Steiner B., Hartl L., Griesser M., Angerer N., Lengauer D., Miedaner T., Schneider B., Lemmens M. (2003). Molecular mapping of QTLs for Fusarium head blight resistance in spring wheat. II. Resistance to fungal penetration and spread. Theor. Appl. Genet..

[31] Buerstmayr H., Stierschneider M., Steiner B., Lemmens M., Griesser M., Nevo E., Fahima T. (2003). Variation for resistance to head blight caused by *Fusarium graminearum* in wild emmer (*Triticum dicoccoides*) originating from Israel. Euphytica.

[32] Steiner B., Buerstmayr M., Wagner C., Danler A., Eshonkulov B., Ehn M., Buerstmayr H. (2019). Fine-mapping of the Fusarium head blight resistance QTL Qfhs.ifa-5A identifies two resistance QTL associated with anther extrusion. Theor. Appl. Genet..

[33] Lemmens M., Scholz U., Berthiller F., Dall’Asta C., Koutnik A., Schuhmacher R., Adam G., Buerstmayr H., Mesterházy Á., Krska R. (2005). The ability to detoxify the mycotoxin deoxynivalenol colocalizes with a major quantitative trait locus for Fusarium head blight resistance in wheat. Mol. Plant-Microbe Interact..

[34] Brown D.W., Butchko R.A.E., Proctor R.H. (2006). Fusarium genomic resources: Tools to limit crop diseases and mycotoxin contamination. Mycopathologia.

[35] Mesterházy Á. (2002). Role of deoxynivalenol in aggressiveness of *Fusarium graminearum* and *F. culmorum* and in resistance to Fusarium head blight. Mycotoxins in Plant Disease.

[36] Mendes G.D.R.L., Ponte E.M.D., Feltrin A.C., Badiale-Furlong E., Oliveira A.C.D. (2018). Common resistance to Fusarium head blight in Brazilian wheat cultivars. Sci. Agric..

[37] Mesterhazy A., Bartók T., Kászonyi G., Varga M., Tóth B., Varga J. (2005). Common resistance to different Fusarium spp. causing Fusarium head blight in wheat. Eur. J. Plant Pathol..

[38] Miedaner T., Heinrich N., Schneider B., Oettler G., Rohde S., Rabenstein F. (2004). Estimation of deoxynivalenol (DON) content by symptom rating and exoantigen content for resistance selection in wheat and triticale. Euphytica.

[39] Proctor R.H., Desjardins A.E., McCornick S.P., Plattner R.D., Alexander N.J., Brown D.W. (2002). Genetic analysis of the role of Trichothecene and Fumonisin mycotoxins in the virulence of Fusarium. Eur. J. Plant Pathol..

[40] Lancova K., Hajslova J., Poustka J., Krplova A., Zachariasova M., Dostálek P., Sachambula L. (2008). Transfer of Fusarium mycotoxins and ‘masked’deoxynivalenol (deoxynivalenol-3-glucoside) from field barley through malt to beer. Food Addit. Contam..

[41] Janick J. (2008). Plant Breeding Reviews.

[42] Steiner B., Buerstmayr M., Michel S., Schweiger W., Lemmens M., Buerstmayr H. (2017). Breeding strategies and advances in line selection for Fusarium head blight resistance in wheat. Trop. Plant Pathol..

[43] Majumdar R., Rajasekaran K., Cary J.W. (2017). RNA interference (RNAi) as a potential tool for control of mycotoxin contamination in crop plants: Concepts and considerations. Front. Plant Sci..

[44] Baulcombe D. (2004). RNA silencing in plants. Nature.

[45] Watson J.M., Fusaro A.F., Wang M., Waterhouse P.M. (2005). RNA silencing platforms in plants. Febs Lett..

[46] Small I. (2007). RNAi for revealing and engineering plant gene functions. Curr. Opin. Biotechnol..

[47] Koch A., Kogel K.H. (2014). New wind in the sails: Improving the agronomic value of crop plants through RNAi-mediated gene silencing. Plant Biotechnol. J..

[48] Nunes C.C., Dean R.A. (2012). Host-induced gene silencing: A tool for understanding fungal host interaction and for developing novel disease control strategies. Mol. Plant Pathol..

[49] Yin C., Scot H.H. (2018). Host-induced gene silencing (HIGS) for elucidating Puccinia gene function in wheat. Plant Pathogenic Fungi and Oomycetes.

[50] Machado A.K., Brown N.A., Urban M., Kanyuka K., Hammond-Kosack K.E. (2018). RNAi as an emerging approach to control Fusarium head blight disease and mycotoxin contamination in cereals. Pest Manag. Sci..

[51] Yin C., Jurgenson J.E., Hulbert S.H. (2011). Development of a host-induced RNAi system in the wheat stripe rust fungus *Puccinia striiformis* f. sp. tritici. Mol. Plant-Microbe Interact..

[52] Cheng W., Song X.S., Li H.P., Cao L.H., Sun K., Qiu X.L., Xu Y.B., Yang P., Huang T., Zhang J.B. (2015). Host-induced gene silencing of an essential chitin synthase gene confers durable resistance to Fusarium head blight and seedling blight in wheat. Plant Biotechnol. J..

[53] Low L.Y., Yang S.K., Andrew Kok D.X., Ong-Abdullah J., Tan N.P., Lai K.S. (2018). Transgenic plants: Gene constructs, vector and transformation method. New Vis. Plant Sci..

[54] Harwood W.A., Hardon J., Ross S.M., Fish L., Smith J., Snape J.W. (2000). Analysis of transgenic barley in a small scale field trial. John Innes Cent. Sainsbury Lab. Annu. Rep..

[55] Romano A., Raemakers K., Visser R., Mooibroek H. (2001). Transformation of potato (*Solanum tuberosum*) using particle bombardment. Plant Cell Rep..

[56] Morikawa H., Sakamoto A., Hokazono H., Irifune K., Takahashi M. (2002). Mechanism of transgene integration into a host genome by particle bombardment. Plant Biotechnol..

[57] Bode J., Benham C., Knopp A., Mielke C. (2000). Transcriptional augmentation: Modulation of gene expression by scaffold/matrix-attached regions (S/MAR elements). Crit. Rev. Eukaryot. Gene. Expr..

[58] Haber J.E. (2000). Partners and pathways: Repairing a double-strand break. Trends Genet..

[59] Gelvin S.B. (2000). *Agrobacterium* and plant genes involved in T-DNA transfer and integration. Annu. Rev. Plant Physiol. Plant Mol. Biol..

[60] Ward D.V., Zambryski P.C. (2001). The six functions of *Agrobacterium* VirE2. Proc. Natl. Acad. Sci. USA.

[61] Gelvin S.B. (2003). *Agrobacterium*-mediated transformation of germinating seeds of *Arabidopsis thaliana*: A non-tissue culture approach. Mol. Gen. Genet..

[62] Zhang Z., Liu X., Wang X., Zhou M., Zhou X., Ye X., Wei X. (2012). An R2R3 MYB transcription factor in wheat, Ta PIMP 1, mediates host resistance to *Bipolaris sorokiniana* and drought stresses through regulation of defense-and stress-related genes. New Phytol..

[63] Liu W., Frick M., Huel R., Nykiforuk C.L., Wang X., Gaudet D.A., Eudes F., Conner R.L., Kuzyk A., Chen Q. (2014). The stripe rust resistance gene Yr10 encodes an evolutionary-conserved and unique CC–NBS–LRR sequence in wheat. Mol. Plant.

[64] Gao L., Wang S., Li X.Y., Wei X.J., Zhang Y.J., Wang H.Y., Liu D.Q. (2015). Expression and functional analysis of a pathogenesis-related protein 1 gene, TcLr19PR1, involved in wheat resistance against leaf rust fungus. Plant Mol. Biol. Report..

[65] Jha S., Chattoo B.B. (2010). Expression of a plant defensin in rice confers resistance to fungal phytopathogens. Transgenic Res..

[66] Li X., Zhong S., Chen W., Fatima S., Huang Q., Li Q., Tan F., Luo P. (2018). Transcriptome analysis identifies a 140 kb region of chromosome 3B containing genes specific to Fusarium Head Blight resistance in wheat. Int. J. Mol. Sci..

[67] Ye X., Cheng H., Xu H., Du L., Lu W., Huang Y. (2005). Development of transgenic wheat plants with chitinase and β-1, 3-glucosanase genes and their resistance to fusarium head blight. Zuo Wu Xue Bao.

[68] Ishida Y., Tsunashima M., Hiei Y., Komari T. (2015). Wheat (*Triticum aestivum* L.) transformation using immature embryos. Agrobacterium Protocols.

[69] San Filippo J., Sung P., Klein H. (2008). Mechanism of eukaryotic homologous recombination. Annu. Rev. Biochem..

[70] Lieber M.R. (2010). The mechanism of double-strand DNA break repair by the nonhomologous DNA end-joining pathway. Annu. Rev. Biochem..

[71] Chapman J.R., Taylor M.R., Boulton S.J. (2012). Playing the end game: DNA double-strand break repair pathway choice. Mol. Cell.

[72] Jiang F., Doudna J.A. (2017). CRISPR–Cas9 structures and mechanisms. Annu. Rev. Biophys..

[73] Matsu-ura T., Baek M., Kwon J., Hong C. (2015). Efficient gene editing in *Neurospora crassa* with CRISPR technology. Fungal Biol. Biotechnol..

[74] Fuller K.K., Chen S., Loros J.J., Dunlap J.C. (2015). Development of the CRISPR/Cas9 system for targeted gene disruption in *Aspergillus fumigatus*. Eukaryot. Cell.

[75] Nødvig C.S., Nielsen J.B., Kogle M.E., Mortensen U.H. (2015). A CRISPR-Cas9 system for genetic engineering of filamentous fungi. PLoS ONE.

[76] Pohl C., Kiel J.A.K.W., Driessen A.J.M., Bovenberg R.A.L., Nygard Y. (2016). CRISPR/Cas9 based genome editing of *Penicillium chrysogenum*. ACS Synth. Biol..

[77] Wenderoth M., Pinecker C., Voß B., Fischer R. (2017). Establishment of CRISPR/Cas9 in *Alternaria alternata*. Fungal Genet. Biol..

[78] Arazoe T., Miyoshi K., Yamato T., Ogawa T., Ohsato S., Arie T., Kuwata S. (2015). Tailor-made CRISPR/Cas system for highly efficient targeted gene replacement in the rice blast fungus. Biotechnol. Bioeng..

[79] Schuster M., Schweizer G., Reissmann S., Kahmann R. (2016). Genome editing in *Ustilago maydis* using the CRISPR–Cas system. Fungal Genet. Biol..

[80] Gardiner D.M., Kazan K. (2018). Selection is required for efficient Cas9-mediated genome editing in *Fusarium graminearum*. Fungal Biol..

[81] Koch A., Hofle L., Werner B.T., Imani J., Schmidt A., Jelonek L., Kogel K.-H. (2019). SIGS vs. HIGS: A study on the efficacy of two dsRNA delivery strategies to silence *Fusarium* FgCYP51 genes in infected host and non-host plants. Mol. Plant Pathol..

[82] He F., Zhang R., Zhao J., Qi T., Kang Z., Guo J. (2019). Host-induced silencing of *Fusarium graminearum* genes enhances the resistance of *Brachypodium distachyon* to Fusarium head blight. Front. Plant Sci..

[83] Su Z., Amy B., Bin T., Hui C., Shan W., Hongxiang M., Shibin C., Liu D., Zhang D., Li T. (2019). A deletion mutation in TaHRC confers Fhb1 resistance to Fusarium head blight in wheat. Nat. Gen..

[84] Mandalà G., Tundo S., Francesconi S., Gevi F., Zolla L., Ceoloni C., D’Ovidio R. (2019). Deoxynivalenol detoxification in transgenic wheat confers resistance to Fusarium head blight and crown rot diseases. Mol. Plant Microbe Interact..

[85] Koch A., Kumar N., Weber L., Keller H., Imani J., Kogel K.H. (2013). Host-induced gene silencing of cytochrome P450 lanosterol C14 alpha-demethylase-encoding genes confers strong resistance to *Fusarium* species. Proc. Natl. Acad. Sci. USA.

[86] McLaughlin J.E., Darwish N.I., Garcia-Sanchez J., Tyagi N., Trick H.N., McCormick S., Dill-Macky R., Tumer N.E. (2021). A lipid transfer protein has antifungal and antioxidant activity and suppresses Fusarium head blight disease and DON accumulation in transgenic wheat. Phytopathology.

[87] Wang M., Wu L., Mei Y., Zhao Y., Ma Z., Zhang X., Chen Y. (2020). Host-induced gene silencing of multiple genes of *Fusarium graminearum* enhances resistance to Fusarium head blight in wheat. Plant Biotechnol. J..

[88] Kumar J., Rai K.M., Pirseyedi S., Elias E.M., Xu S., Dill-Macky R., Kianian S.F. (2020). Epigenetic regulation of gene expression improves Fusarium head blight resistance in durum wheat. Sci. Rep..

[89] Gatti M., Florence C., Caroline T., Catherine M., Florence G., Thierry L., Marie D. (2019). The *Brachypodium distachyon* UGT Bradi5gUGT03300 confers type II fusarium head blight resistance in wheat. Plant Pathol..

[90] Li G., Jiyang Z., Haiyan J., Zhongxia G., Min F., Yanjun L., Panting Z., Xue S., Li N., Yuan Y. (2019). Mutation of a histidine-rich calcium-binding-protein gene in wheat confers resistance to Fusarium head blight. Nat. Gen..

[91] Mandalà G., Carla C., Isabella B., Francesco F., Silvio T. (2021). Transgene pyramiding in wheat: Combination of deoxynivalenol detoxification with inhibition of cell wall degrading enzymes to contrast Fusarium Head Blight and Crown Rot. Plant Sci..

[92] Hu W., Gao D., Wu H., Liu J., Zhang C., Wang J., Jiang Z., Liu Y., Li D., Zhang Y. (2020). Genome-wide association mapping revealed syntenic loci QFhb-4AL and QFhb-5DL for Fusarium head blight resistance in common wheat (*Triticum aestivum* L.). BMC Plant Biol..

[93] Słomińska-Durdasiak K.M., Sonja K., Viktor K., Daniela N., Patrick S., Armin D., Jochen C.R. (2020). Association mapping of wheat Fusarium head blight resistance-related regions using a candidate-gene approach and their verification in a biparental population. Theor. Appl. Genet..

[94] Tessmann E.W., Dong Y., Van Sanford D.A. (2019). GWAS for Fusarium head blight traits in a soft red winter wheat mapping panel. Crop Sci..

[95] Sonia E., Dorothée S., Sandrine G., Corinne C., Christian L., Henri L.M., Valérie L. (2018). Optimized real time QPCR assays for detection and quantification of *Fusarium* and *Microdochium species* involved in wheat head blight as defined by MIQE guidelines. BioRxiv.

[96] Burlakoti R.R., Estrada Jr R., Rivera V.V., Boddeda A., Secor G.A., Adhikari T.B. (2007). Real-time PCR quantification and mycotoxin production of *Fusarium graminearum* in wheat inoculated with isolates collected from potato, sugar beet, and wheat. Phytopathology.

[97] Munis M.F.H., Xu S., Hakim H.J.C., Masood S., Farooq A.B.U. (2018). Diagnosis of *Fusarium graminearum* in Soil and Plant Samples of Wheat by Real-Time PCR. Rom. Biotechnol. Lett..

[98] Tralamazza S.M., Braghini R., Corrêa B. (2016). Trichothecene genotypes of the *Fusarium graminearum* species complex isolated from Brazilian wheat grains by conventional and quantitative PCR. Front. Microbiol..

[99] Gatti M., Choulet F., Macadré C., Guérard F., Seng J.M., Langin T., Dufresne M. (2018). Identification, molecular cloning and functional characterization of a wheat UDP-glucosyltransferase involved in resistance to Fusarium Head Blight and to mycotoxin accumulation. Front. Plant Sci..

